# Incident HFpEF and time-dependent changes in markers of LVDD severity in women and men with preclinical LVDD

**DOI:** 10.1136/openhrt-2024-003105

**Published:** 2025-05-04

**Authors:** Anne Margje Lisa Naomi van Ommen, Elisa Dal Canto, Ernest Diez Benavente, Maarten Jan Cramer, Arco J Teske, Roxana Menken, Karim Taha, M Louis Handoko, Dirk J Duncker, Marianne C Verhaar, Frans H Rutten, N Charlotte Onland-Moret, Hester M den Ruijter

**Affiliations:** 1 Laboratory of Experimental Cardiology, University Medical Center, Utrecht University, Utrecht, The Netherlands; 2 Julius Center for Health Sciences and Primary Care, University Medical Center, Utrecht, The Netherlands; 3 Department of Cardiology, University Medical Center, Utrecht University, Utrecht, The Netherlands; 4 Cardiology Centers of the Netherlands, Utrecht, The Netherlands; 5 Department of Cardiology, Amsterdam University Medical Center, Amsterdam, The Netherlands; 6 Department of Cardiology, Erasmus University Medical Center, Rotterdam, The Netherlands; 7 Department of Nephrology and Hypertension, University Medical Center Utrecht, Utrecht University, Utrecht, The Netherlands

**Keywords:** DIASTOLIC DYSFUNCTION, Echocardiography, EPIDEMIOLOGY, RISK FACTORS

## Abstract

**Background:**

The progression of left ventricular diastolic dysfunction (LVDD) over time may lead to the development of heart failure with preserved ejection fraction (HFpEF). HFpEF is twice as common in women compared with men; however, the sex-specific progression from LVDD towards HFpEF is poorly described. Therefore, we aim to evaluate changes over time in markers of LVDD severity and HFpEF in women and men with preclinical LVDD.

**Methods and results:**

We reassessed 146 participants from the HELPFul study (58% women and 42% men) with preclinical LVDD after a median follow-up of 4.3 (IQR: 3.9–4.7) years. The follow-up measurements mirrored baseline measurements, encompassing clinical examination, blood draw for biomarkers and echocardiography. We determined HFpEF incidence and report changes over time in echocardiography. Additionally, we studied how blood pressure and kidney function affected LVDD progression, including plasma N-terminal pro-B-type natriuretic peptide (NT-proBNP) levels, using generalised mixed models. All analyses were performed for women and men combined, and sex stratified. Out of 146 participants, 15 (10%) developed HF of whom 13 had HFpEF (9 women and 4 men). Over time, mean kidney function (estimated glomerular filtration rate, eGFR) declined from 89±14.4 to 81±16.9 mL/min/1.73 m^2^ and median NT-proBNP plasma levels increased from 71 (IQR: 44–120) to 100 (IQR: 51–157) pg/mL. In women, a higher systolic and in men a higher diastolic blood pressure were associated with an increase in NT-proBNP plasma levels over time. Lower eGFR levels were related to increased NT-proBNP plasma levels over time in both men and women.

**Conclusions:**

Our study demonstrates that only a small proportion of women and men with preclinical LVDD develop incident HF over a roughly 5-year follow-up period. High blood pressure and decreased kidney function were associated with higher levels of NT-proBNP. This highlights the need to further explore cardiorenal protection as a method to prevent HFpEF.

**WHAT IS ALREADY KNOWN ON THIS TOPIC**

- Approximately one-quarter of adults in the general population are affected by left ventricular diastolic dysfunction (LVDD), and the prevalence of LVDD doubles every 10 years in individuals aged 45 years and older. A proportion of patients will develop heart failure with preserved ejection fraction (HFpEF) over time, but longitudinal studies on this are sparse. Therefore, the management of individuals with LVDD but without symptoms remains challenging as current guidelines recommend treating comorbidities associated with LVDD, without providing specifics on medical interventions or follow-up.

**WHAT THIS STUDY ADDS**

- This is the first longitudinal study investigating the progression from LVDD without symptoms towards HFpEF for women and men separately. Additionally, this is the first study to provide high-quality echocardiography data at the two time points, stratified by sex.

**HOW THIS STUDY MIGHT AFFECT RESEARCH, PRACTICE OR POLICY**

- This study found a relatively low annual incidence rate of HFpEF of approximately 2%. Additionally, there were no marked changes in echocardiography over time. Therefore, routine echocardiography follow-up in LVDD patients for early detection of HF may not be feasible nor recommended.

## Introduction

 Left ventricular diastolic dysfunction (LVDD) is a condition characterised by impaired LV relaxation and/or increased LV passive stiffness, potentially leading to elevated LV filling pressures.^1^ Notably, LVDD is a risk factor for both cardiovascular and all-cause mortality, underscoring its clinical significance.^2^ The progression of LVDD over time may lead to the development of heart failure with preserved ejection fraction (HFpEF).^3^ Interestingly, HFpEF is twice as common in women compared with men, despite the prevalence of LVDD being similar between the two sexes.^3^ However, to date, longitudinal studies on the sex-specific progression of LVDD towards HFpEF are not available. Additionally, the few available studies on the progression of LVDD towards HF lack repeated echocardiography and biomarker measurements, and details on HF subtypes.

Echocardiography has a pivotal role in the evaluation of LVDD, which requires the assessment of multiple functional and morphological markers. Ageing strongly influences these markers. Approximately one-quarter of adults in the general population are affected by LVDD, and the prevalence of LVDD doubles every 10 years in individuals aged 45 years and older.^4^ Nevertheless, the management of individuals with LVDD but without symptoms remains challenging as current guidelines recommend treating comorbidities associated with LVDD, without providing specifics on medical interventions or follow-up.^5 6^ Arterial hypertension is a well-established risk factor for LVDD,^7^ and kidney impairment has more recently emerged as an important additional risk factor for LVDD.^8 9^ However, the extent to which blood pressure and kidney function contribute to the progression of LVDD is poorly described. Recognising LVDD at an early stage can facilitate preventive measures aimed at halting disease progression. Therefore, gaining a comprehensive understanding of LVDD progression is essential. We aim to determine the incidence of HFpEF and progression in markers of LVDD severity in a well-phenotyped cohort of patients with preclinical LVDD.

## Methods

### Study population

**Figure 1:**
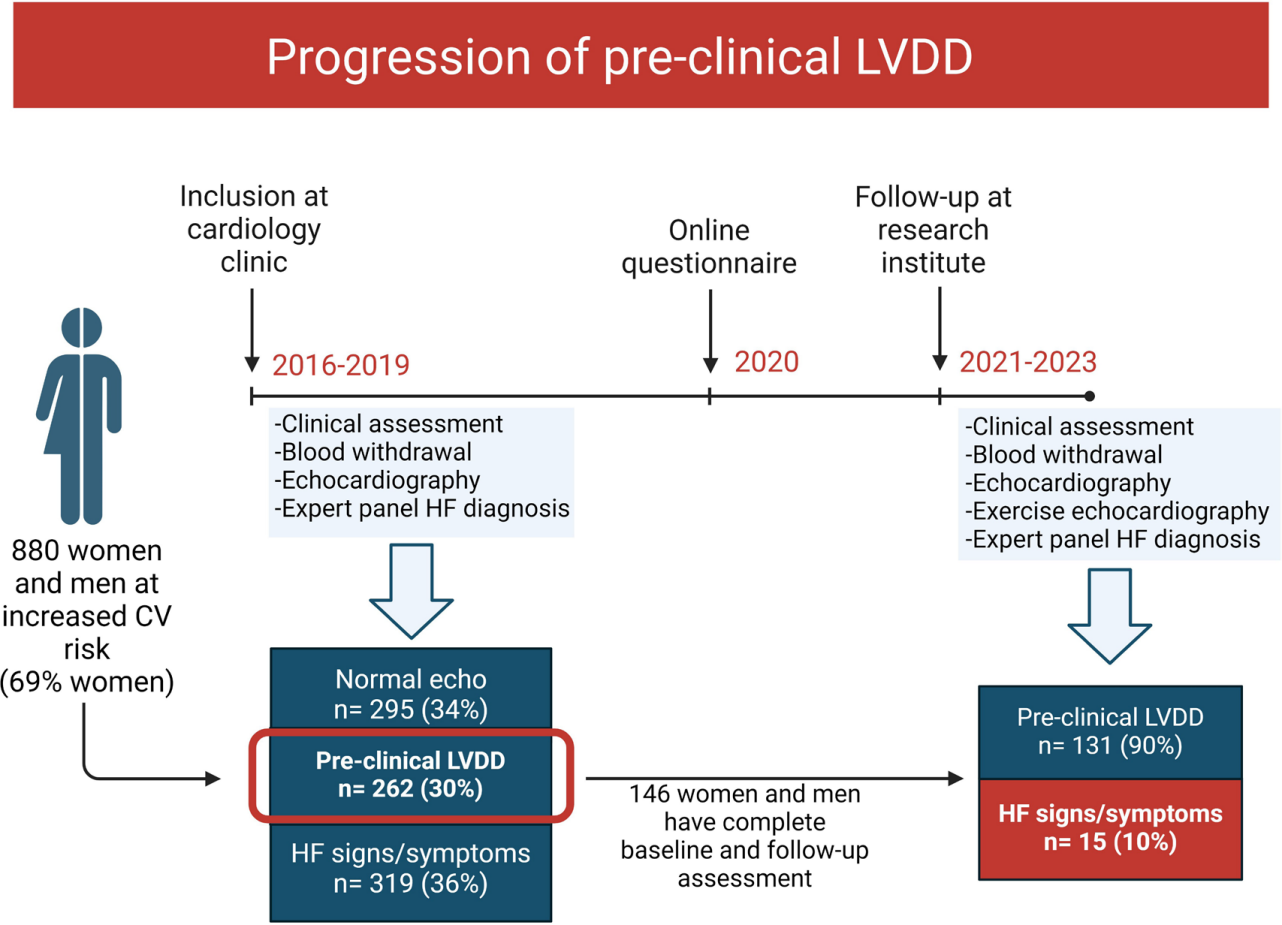
Description of study procedures and selection of eligible participants for follow-up assessment. The HELPFul cohort consists of 880 patients at increased cardiovascular risk who were referred to outpatient cardiology clinics for cardiovascular assessment. From all patients in the HELPFul cohort, a total of 146 patients diagnosed with preclinical LVDD at baseline participated in the follow-up study, and 15 patients (10.3%) developed overt HF. The majority of patients developed HFpEF (n=13) and were female (n=10). CV, cardiovascular; HF, heart failure; LVDD, left ventricular diastolic dysfunction.

The HELPFul study served as the base for this study.^10^ In brief, HELPFul is a cohort study that enrolled participants ≥45 years at increased cardiovascular risk, referred for cardiac evaluation. A total of 880 participants were recruited. Among these participants, 262 individuals (30% of the total cohort) exhibited preclinical LVDD, indicating that HF symptoms were absent. These participants were eligible for the HELPFulUP study. Only those participants who provided explicit consent to be contacted for further research (n=213) were invited. The assessment involved repeated high-quality echocardiographic measurements of diastolic function (figure 1). A total of 146 participants consented to participate and were included for follow-up at the research institute (see flow chart in online supplemental figure 1 for non-participation reasons).

### HF definition

Similar to baseline, an expert panel determined the likelihood of LVDD, and/or HFpEF or HF with reduced ejection fraction (HFrEF) based on the clinical presentation, diagnostic results and guidelines.^10^ HFpEF and HFrEF were established when signs or symptoms of HF were present along with echocardiographic abnormalities. HFrEF was diagnosed when LV ejection fraction was below 50%.

### Assessment of clinical parameters

Information on medical history, lifestyle habits and medication use was systematically gathered at baseline and follow-up. Systolic and diastolic blood pressure (SBP and DBP) were measured at rest following protocol. Antihypertensive medication included ACE inhibitors, angiotensin-receptor blockers, beta-blockers, calcium-channel blockers, thiazide diuretics and aldosterone receptor antagonists.

### Blood biomarker assessment

Venous blood samples for plasma cardiac biomarkers and biobank purposes were collected at both time points. N-terminal pro-B-type natriuretic peptide (NT-proBNP) at baseline and creatinine, cystatin C and glycated haemoglobin were measured with the ARCHITECT i2000 analyser (Abbott Park, Chicago, Illinois, USA). At follow-up, NT-proBNP was measured using the Atellica Immunoassay Analyzer (Siemens, USA). NT-proBNP measurements are comparable across platforms.^11 12^ Kidney function was estimated according to the Chronic Kidney Disease Epidemiology Collaboration (CKD-EPI 2021) equation resulting in an estimated glomerular filtration rate (eGFR, mL/min/1.73 m^2^) based on a combination of creatinine and cystatin C levels. We also calculated eGFR according to the CKD-EPI equation based on creatinine alone for comparison.

### Echocardiography and outcome assessment

At baseline, participants underwent rest echocardiography using a standardised protocol and General Electric Vivid E6 or E7 ultrasound device (GE, Horten, Norway). At follow-up, additional exercise echocardiography was performed using a GE Vivid9 ultrasound machine.^13^ Key parameters included E and A wave velocity and pulsed-wave TDI lateral and septal e′ velocities to calculate the E/A and E/e’ ratio, tricuspid regurgitation (TR) signal, LV mass index (LVMI) and left atrial volume index (LAVI). For full (exercise) echocardiography details, we refer to online supplemental methods 1.

In our analyses, we used three outcomes assessed at baseline and follow-up based on the Heart Failure Association (HFA) how to diagnose HFpEF algorithm (HFA-PEFF diagnostic algorithm).^14^ Outcomes were log transformed NT-proBNP, covering the biomarker part of the HFA-PEFF score, reflecting cardiac wall stress and the presence of major functional and major morphological abnormalities. Major functional abnormalities included septal and lateral e’ velocity below 7 and 10 cm/s, respectively, an average E/e’ ratio ≥15 or a TR velocity >280 cm/s. Major morphological abnormalities included LAVI>34 mL/m² or concentric hypertrophy (relative wall thickness >0.42 and LVMI≥149 g/m² in men or ≥122 g/m² in women). The absence of any abnormalities, along with minor abnormalities, served as the reference group.

### Statistical analysis

We assessed associations between each determinant (fixed effects) and changes in log NT-proBNP (continuously), and major functional or morphological abnormalities according to the HFA-PEFF algorithm (binary) over time, using linear and logistic mixed-effects models (depending on the outcome). For these analyses, we included a time variable capturing the longitudinal aspect of the data and a random intercept to account for individual repeated measures. The determinants of interest were SBP, DBP and eGFR per SD change. Crude associations and associations adjusted for relevant confounders were tested. Models with kidney function as a determinant were not adjusted for age since age is part of the equation to calculate kidney function. All models were conducted for women and men separately, and we tested effect modification by sex in the combined dataset of women and men using an interaction term. Finally, we explored whether the models’ performance improved by adding an interaction term for time and each determinant. If such improvement was observed, a separate effect estimate for the change in determinant was reported. For continuous outcomes, we present beta with their corresponding 95% CI, and for binary outcomes, ORs and 95% CIs. Missing values were present in a proportion ranging from 0.8% for smoking to 38.4% for eGFR at follow-up (online supplemental table 1) and were imputed using multiple imputation with the mice package. We used R-Studio V.4.2.3. for data analysis. A p<0.05 was considered statistically significant.

## Results

The baseline characteristics of the study base, comprising 880 individuals, are presented in online supplemental table 2. As per design, the HELPFul cohort was representative of a high-risk cardiovascular population in the Netherlands visiting outpatient clinics.

### Baseline characteristics of the 146 patients with preclinical LVDD

At baseline, the average age was 63 (±SD 9) years, and 58% were women (table 1). Average body mass index was 26.7 kg/m^2^ (±SD 4.3), and 58% and 6% of patients reported hypertension or diabetes, respectively. On the day of inclusion, 35% of patients used blood pressure medication, but these were less often prescribed in women than men (32% compared with 39%). The average eGFR was 89 (±SD 14) mL/min/1.73 m^2^, with women showing slightly lower eGFR compared with men.

**Table 1:** Clinical characteristics at baseline and follow-up

n	Overall		Men		Women	
	146		61		85	
	Baseline	Follow-up	Baseline	Follow-up	Baseline	Follow-up
Age, years, mean (±SD)	63 (9)	67 (9)	63 (9)	67 (9)	63 (8)	67 (8)
BMI, kg/m^2^, mean (±SD)	26.7 (4.3)	27.4 (4.6)	27.6 (4.1)	27.7 (4.2)	26.1 (4.5)	27.1 (4.9)
Obesity, n (%)	28 (20)	38 (26)	11 (19)	17 (28)	17 (21)	21 (25)
SBP, mm Hg, mean (±SD)	146 (19)	145 (20)	149 (21)	148 (19)	144 (17)	142 (20)
DBP, mm Hg, mean (±SD)	89 (11)	85 (13)	91 (11)	84 (12)	87 (10)	85 (13)
Creatinine, mmol/L, mean (±SD)	71 (12)	80 (14)	78 (12)	87 (12)	67 (10)	73 (13)
eGFR (CKD-EPI), mL/min/1.73 m^2^, mean (±SD)	90 (12)	82 (14)	94 (11)	85 (14)	88 (12)	79 (14)
eGFR (CKD-EPI including cystatin C), mL/min/1.73 m^2^, mean (±SD)	89 (14)	81 (17)	91 (14)	83 (17)	88 (15)	78 (17)
Proportion of predicted workload achieved, mean (±SD)	0.87 (0.20)	1.01 (0.19)	0.92 (0.21)	0.96 (0.09)	0.84 (0.19)	1.04 (0.24)
Self-reported hypertension, n (%)	85 (58)	83 (56.8)	40 (66)	38 (62)	45 (53)	45 (53)
Hypercholesterolaemia, n (%)	65 (45)	65 (45)	27 (44)	33 (54)	38 (45)	32 (38)
Diabetes, n (%)	8 (6)	10 (7)	5 (8)	7 (12)	3 (4)	3 (4)
HbA1c, mmol/mol, mean (±SD)	36 (6)	37 (6)	37 (7)	37 (6)	36 (5)	37 (6)
Atrial fibrillation, n (%)	4 (3)	13 (9)	3 (5)	6 (10)	1 (1)	7 (8)
Ischaemic heart disease, n (%)	17 (12)	20 (14)	12 (20)	13 (21)	5 (6)	7 (8)
Alcohol consumption, n (%)						
No	9 (7)	13 (9)	3 (5)	3 (5)	6 (8)	10 (13)
Not daily	64 (47)	65 (47)	23 (39)	24 (41)	41 (53)	41 (52)
Daily	64 (47)	60 (44)	33 (56)	32 (54)	31 (40)	28 (35)
Smoking, n (%)						
Never	59 (41)	59 (41)	25 (42)	25 (42)	34 (41)	34 (41)
Current	11 (8)	7 (5)	6 (10)	4 (7)	5 (6)	3 (4)
Former	74 (51)	78 (54)	29 (48)	31 (52)	45 (54)	47 (56)
Any antihypertensive use, n (%)	51 (35)	76 (52)	24 (39)	35 (57)	27 (32)	41 (48)
Beta-blockers	14 (10)	22 (15)	7 (12)	14 (23)	7 (8)	8 (9)
ACE-inhibitors	15 (10)	17 (12)	8 (13)	7 (12)	7 (8)	10 (12)
ARBs	20 (14)	39 (27)	11 (18)	17 (28)	9 (11)	22 (26)
CCBs	12 (8)	25 (17)	5 (8)	11 (18)	7 (8)	14 (17)
Thiazide diuretics	19 (13)	20 (14)	5 (8)	8 (13)	14 (17)	12 (14)
Statins, n (%)	37 (25)	58 (40)	17 (28)	29 (48)	20 (24)	29 (34)
Hypoglycaemic agents, n (%)	9 (6)	6 (5)	4 (7)	6 (10)	1 (1)	3 (4)

ARBs, angiotensin receptor blockers; BMI, body mass index; CCB, calcium-channel blockers; CKD-EPI, Chronic Kidney Disease Epidemiology Collaboration; DBP, diastolic blood pressure; eGFR, estimated glomerular filtration rate; EPI, Epidemiology Collaboration; HbA1c, glycated haemoglobin; SBP, systolic blood pressure.

### HF incidence

Over the 4.3 years (IQR: 3.9–4.7) of follow-up, a total of 15 patients developed HF, of whom the majority developed HFpEF (n=13) (table 2). Specifically, nine women (11%) and four men (7%) developed HFpEF (p=0.56). One woman and one man developed HFrEF. Based on these findings, the annual incidence of HFpEF is 2% in this cohort. The characteristics of the individuals who developed HFpEF are in online supplemental table 3.

**Table 2:** Markers of LVDD severity at baseline and follow-up

	Overall (n=146)		Men (n=61)		Women (n=85)		Comparison by sex	
	Baseline	Follow-up	Baseline	Follow-up	Baseline	Follow-up	P value baseline	P value follow-up
NT-proBNP, pg/mL, median (25th quartile, 75th quartile)	71 (44, 120)	100 (51, 157)	54 (30, 112)	78 (39, 150)	82 (51, 124)	113 (61, 157)	0.05	0.08
Functional abnormalities HFA-PEFF algorithm, n (%)								
Absent	13 (9)	4 (3)	4 (7)	1 (2)	9 (11)	3 (4)	0.37	0.07
Minor	10 (7)	9 (6)	6 (10)	7 (12)	4 (5)	2 (2)		
Major	117 (84)	133 (91)	49 (83)	53 (87)	68 (84)	80 (94)		
Morphological abnormalities HFA-PEFF algorithm, n (%)							0.007	0.28
Absent	30 (21)	33 (23)	7 (12)	10 (16)	23 (27)	23 (27)		
Minor	80 (55)	56 (38)	32 (53)	24 (39)	48 (57)	32 (38)		
Major	36 (25)	57 (39)	22 (36)	27 (44)	14 (17)	30 (35)		
HFpEF, n (%)		13 (9)		4 (7)		9 (11)		0.56
HFrEF, n (%)		2 (1)		1 (2)		1 (1)		1

HFA-PEFF refers to the diagnostic HFpEF algorithm by the Heart Failure Association from the European Society of Cardiology.

HFpEF, heart failure with preserved ejection fraction; HFrEF, heart failure with reduced ejection fraction; LVDD, left ventricular diastolic dysfunction; NT-proBNP, N-terminal pro-brain natriuretic peptide.

### Changes over time in markers of LVDD severity

**Figure 2:**
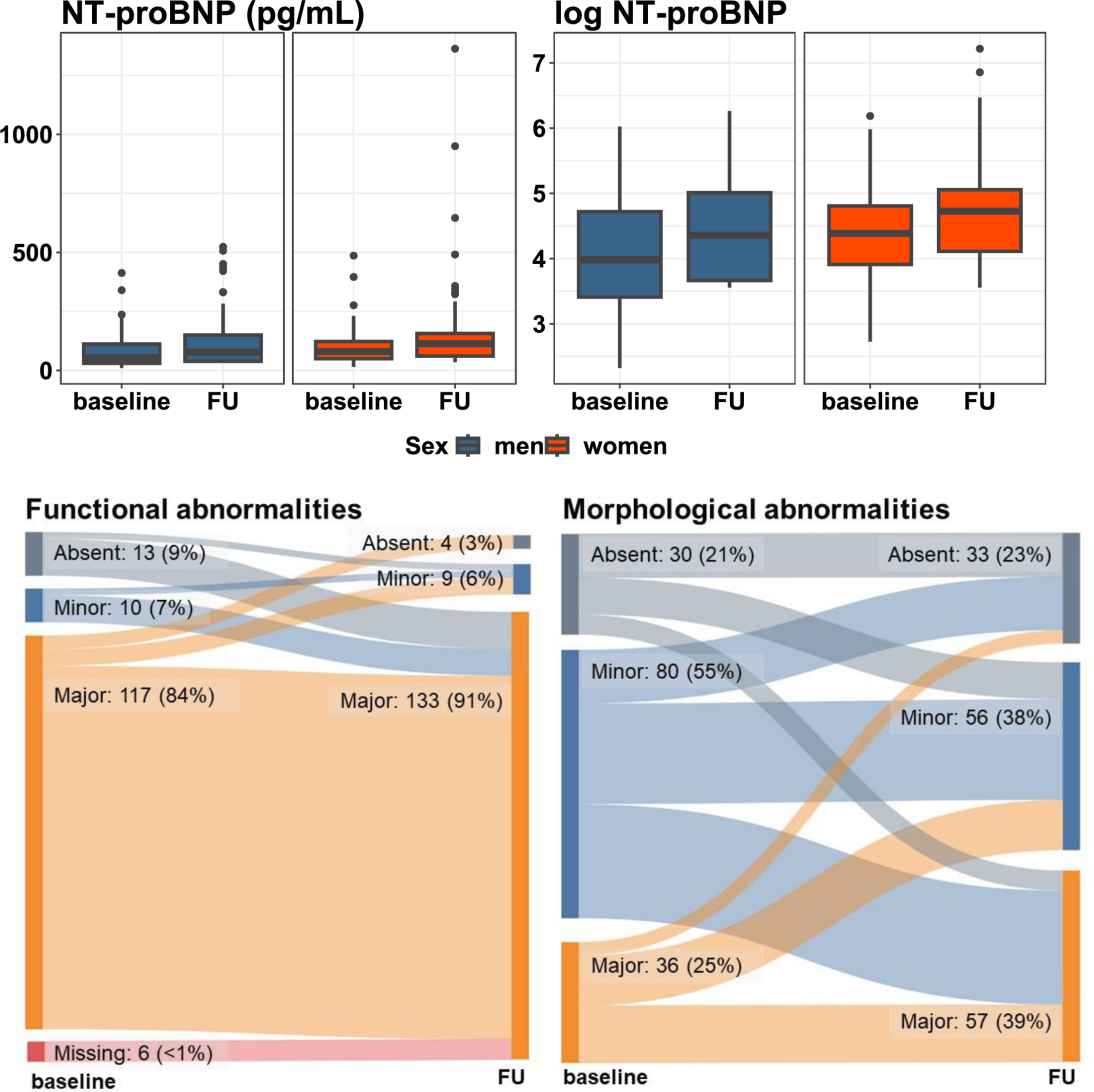
Longitudinal changes in markers of LVDD severity. Boxplots (top) showing change in NT-proBNP and log NT-proBNP over time, comparing women and men. Change in functional and morphological abnormalities according to the HFA-PEFF score from baseline to follow-up is displayed in Sankey plots (bottom). When we study changes over time in functional and morphological abnormalities in logistic mixed models, the absence of any abnormalities, together with minor abnormalities, are grouped as reference, and major abnormalities are the binary outcome. HFA-PEFF, Heart Failure Association from the European Society of Cardiology; NT-proBNP, N-terminal pro-brain natriuretic peptide.

The median NT-proBNP plasma level at baseline was 71 (IQR: 44–120) pg/mL, which increased to 100 (IQR: 51–157) pg/mL at follow-up. Baseline and follow-up levels of NT-proBNP were 82 (IQR: 51–124) and 113 (IQR: 61–157) pg/mL in women and 54 (IQR: 30–112) and 78 (IQR: 39–150) pg/mL in men. However, the difference between women and men was not statistically significant (p=0.05 and 0.08 for between sex comparison at baseline and at follow-up, respectively; table 2 and figure 2). When examining the change in log NT-proBNP over 5 years, a significant rise in NT-proBNP over time was observed (β=0.42 (95% CI: 0.3, 0.45)), which was consistent for women and men (p value_sex interaction_=0.13) (table 3).

**Table 3:** Results of mixed models: the effects of time, blood pressure parameters and kidney function on markers of LVDD severity

		Log NT-proBNP				
		All	Women	Men		
		Beta (95% CI) for change in outcome over time	Beta (95% CI) for change in outcome over time	Beta (95% CI) for change in outcome over time	P value sex interaction	
Time (per 5 years)	Crude	**0.42 (0.3, 0.54**)	**0.41 (0.26, 0.56**)	**0.43 (0.23, 0.63**)	0.13	
		Beta (95% CI)	Beta (95% CI)	Beta (95% CI)	P value sex interaction	P value time-exposure interaction
SBP (per SD increase)	Crude	**0.12 (0.05, 0.20**)	**0.16 (0.06, 0.26**)	0.09 (-0.04, 0.22)	**0.043**	0.14
	Adjusted*	**0.09 (0.02, 0.17**)	**0.13 (0.03, 0.23**)	0.06 (-0.06, 0.19)	0.10	**0.049**
DBP (per SD increase)	Crude	0.01 (-0.07, 0.09)	−0.05 (-0.15, 0.05)	0.09 (-0.05, 0.23)	**0.046**	0.41
	Adjusted*	**0.08 (0.00, 0.15**)	0.02 (-0.08, 0.13)	**0.18 (0.04, 0.31**)	**0.045**	0.41
eGFR (per SD decrease)	Crude	**0.12 (0.01, 0.22**)	0.11 (-0.01, 0.23)	0.11 (-0.06, 0.28)	0.18	0.89
	Adjusted†	**0.12 (0.01, 0.22**)	0.11 (-0.02, 0.24)	0.10 (-0.07, 0.27)	0.26	0.87
		**HFA major functional abnormalities**				
		**All**	**Women**	**Men**		
		OR (95% CI) for change in outcome over time	OR (95% CI) for change in outcome over time	OR (95% CI) for change in outcome over time	P value sex interaction	
Time (per 5 years)	Crude	**2.7 (1.18, 6.18**)	**4.02 (1.04, 15.5**)	1.79 (0.47, 6.81)	0.40	
		OR (95% CI)	OR (95% CI)	OR (95% CI)	P value sex interaction	P value time-exposure interaction
SBP (per SD increase)	Crude	0.87 (0.57, 1.34)	0.74 (0.44, 1.23)	1.08 (0.54, 2.15)	0.50	0.95
	Adjusted*	0.90 (0.59, 1.39)	0.79 (0.46, 1.36)	1.10 (0.57, 2.12)	0.66	0.82
DBP (per SD increase)	Crude	1.06 (0.66, 1.70)	0.82 (0.47, 1.44)	1.42 (0.65, 3.12)	0.33	0.95
	Adjusted*	1.20 (0.71, 2.02)	0.96 (0.49, 1.88)	1.29 (0.57, 2.90)	0.69	0.99
eGFR (per SD decrease)	Crude	0.86 (0.52, 1.42)	1.02 (0.53, 1.96)	0.47 (0.09, 2.51)	0.30	0.93
	Adjusted†	0.80 (0.49, 1.32)	0.95 (0.49, 1.84)	0.58 (0.26, 1.26)	0.76	0.90
		**HFA major morphological abnormalities**				
		**All**	**Women**	**Men**		
		OR (95% CI) for change in outcome over time	OR (95% CI) for change in outcome over time	OR (95% CI) for change in outcome over time	P value sex interaction	
Time (per 5 years)	Crude	**2.09 (1.14, 3.82**)	**2.75 (1.21, 6.28**)	1.63 (0.62, 4.32)	**0.03**	
		OR (95% CI)	OR (95% CI)	OR (95% CI)	P value sex interaction	P value time-exposure interaction
SBP (per SD increase)	Crude	1.03 (0.79, 1.35)	0.89 (0.63, 1.26)	1.11 (0.69, 1.78)	**0.037**	0.16
	Adjusted*	1.02 (0.78, 1.35)	0.83 (0.56, 1.24)	1.05 (0.65, 1.69)	0.32	0.18
DBP (per SD increase)	Crude	1.14 (0.87, 1.49)	0.88 (0.62, 1.26)	1.47 (0.90, 2.41)	**0.012**	0.25
	Adjusted*	1.16 (0.86, 1.56)	0.95 (0.62, 1.44)	1.24 (0.73, 2.10)	0.34	0.26
eGFR (per SD decrease)	Crude	1.00 (0.74, 1.36)	0.93 (0.62, 1.41)	1.23 (0.71, 2.10)	0.051	0.67
	Adjusted†	1.00 (0.73, 1.36)	0.92 (0.58, 1.44)	1.20 (0.70, 2.05)	0.29	0.75

Bold values represent significant associations.

* Adjusted for age, body mass index, diabetes mellitus, hypercholesterolaemia, cardiovascular history, alcohol consumption, smoking status and education level.

† Adjusted for hypertension, body mass index, diabetes mellitus, hypercholesterolaemia, cardiovascular history, alcohol consumption, smoking status and education level.

DBP, diastolic blood pressure; eGFR, estimated glomerular filtration rate; HFA, Heart Failure Association; NT-proBNP, N-terminal pro-brain natriuretic peptide; SBP, systolic blood pressure.

Major functional abnormalities were prevalent at baseline and follow-up (84% and 91%). There were no significant sex differences in the prevalence of functional abnormalities according to the HFA-PEFF algorithm (table 2 and figure 2). Over time, there was a significant rise in the presence of major functional abnormalities per 5 years (OR=2.7 (95% CI: 1.18, 6.18)), which was consistent in both women and men (p value_sex interaction_=0.40) (table 3).

Major morphological abnormalities were generally less common than functional abnormalities, present in 25% of the population at baseline and in 39% at follow-up. At baseline, major morphological abnormalities were significantly more common in men (36%) than women (17%) (p=0.007). However, this difference was no longer present at follow-up (44% in men, 35% in women, p=0.28; table 2 and figure 2). A significant rise in major morphological abnormalities over time was observed (OR=2.09 (95% CI: 1.14, 3.82)), with a stronger effect in women than in men (p_sex interaction_=0.03). In women, the risk of having major morphological abnormalities increased over time (OR=2.75 (95% CI: 1.21, 6.28)), whereas in men the risk was much lower (OR=1.63 (95% CI: 0.62, 4.32)) and not statistically significant (table 3). Baseline and follow-up values of other echocardiographic measurements are presented in online supplemental table 3.

### Associations between blood pressure and kidney function and changes in markers of LVDD severity over time

Subsequently, we investigated the determinants of time-dependent changes in markers of LVDD severity (NT-proBNP and major functional and morphological abnormalities according to the HFA-PEFF algorithm). Increments in SBP, DBP and eGFR at baseline and follow-up combined were significantly associated with higher (log) NT-proBNP over time (table 3). Each SD increase in SBP and DBP led to an increase in log NT-proBNP over time (β=0.09 (95% CI: 0.02, 0.17) and β=0.08 (95% CI: 0.00, 0.15)) after adjustments for confounders. As expected, a decrease in eGFR, indicative of reduced kidney function, resulted in a higher (log) NT-proBNP over time (β=0.12 (95% CI: 0.01, 0.22)) after adjusting for confounders.

When stratifying the analyses by sex, the relationship between SBP and change in (log) NT-proBNP over time was only significant in women (β=0.13 (95% CI: 0.03, 0.23)), with no significant difference from men (p_sex interaction_=0.10). Conversely, the association of DBP with (log) NT-proBNP over time was only significant in men (β=0.18 (95% CI: 0.04, 0.31)), and this was significantly different from the findings in women (p_sex interaction_=0.045).

There were no associations between SBP, DBP or eGFR and change in major functional and morphological abnormalities over time according to the HFA-PEFF algorithm. Additionally, we did not observe significant interaction for sex.

Finally, we investigated whether the models significantly improved by introducing an interaction term between time and exposure. This would imply that a change (worsening) in the exposure value over time increases the risk of worsening in outcome values, in addition to the effect of baseline and follow-up values separately. Only for SBP, we observe that changes in SBP significantly affected changes in NT-proBNP (p=0.049). However, it appeared that a rise in SBP over time led to a reduction in (log) NT-proBNP levels (β=−0.13 (95% CI: −0.27, 0)), contrary to our expectations.

## Discussion

The study employed standardised follow-up of asymptomatic patients with preclinical LVDD. The findings reveal a relatively low annual incidence rate of HFpEF of 2%, alongside limited change in individual echocardiography parameters of LVDD in both women and men over a 5-year follow-up. Additionally, our analyses reveal that impaired kidney function as well as higher blood pressure are associated with a rise in NT-proBNP plasma levels over time.

### Comparing HF incidence

Prior studies have reported a wide range of annual HF incidence in populations with preclinical LVDD, between 1.2% and 10.3%.^3^ Some studies had longer follow-up time than ours but did not distinguish between HFpEF and HFrEF nor report sex-specific data.^3^ In our study, more women (11%) than men (7%) developed HFpEF, aligning with other research showing that HFpEF is more dominant in women than men.^15^

The incidence of HF in our study may differ from other studies due to population differences. At baseline, all participants were screened and treated for cardiovascular issues by their cardiologist. As a result, our population may be relatively well controlled in terms of cardiovascular risk factors compared with cohorts sampled from the general community or databases. Additionally, participants with preclinical LVDD had no HF symptoms at baseline, unlike those in other studies with suggestive signs.^16^ Low prevalence of diabetes, atrial fibrillation, coronary artery disease and obesity underscores effective risk factor management. As a result, disease progression may occur at a slower pace compared with other studies.

### Blood pressure and kidney function

Our study offered the unique opportunity to investigate the course of preclinical LVDD when relatively unaffected by cardiac and systemic comorbidities. We postulated that, aside from ageing, hypertension and kidney dysfunction were the major contributors to diastolic dysfunction. Kidney function, SBP and DBP were associated with a rise in NT-proBNP levels over time. One community-based study found that hypertension medication initiation and decreasing eGFR increased natriuretic peptide levels over 10 years, when adjusting for age and sex.^17^ In our study, from all models, only a rise in SBP with time borderline significantly lowered NT-proBNP. Notably, this finding was directed contrary to our expectations. Potentially, this indicated that patients with higher SBP at baseline, exposed to prolonged periods of elevated blood pressure, experienced a less pronounced rise in NT-proBNP compared with those who had a steeper increase in blood pressure over time. This indicates that blood pressure (treatment) and NT-proBNP are closely connected.^17^,^19^ An impaired kidney function is known to result in decreased excretion of NT-proBNP, which may potentially lead to an overestimation of our findings. However, in our study, most patients had eGFR levels that fall within normal ranges; therefore, we assume that this effect may be negligible, as previously shown by others.^20^ Nevertheless, when comparing baseline and follow-up kidney function, we observed a decline in kidney function that exceeds the expected 1 mL/min/m^2^ per year change, warranting further exploration in future studies.

### Sex differences

When stratifying our analyses by sex, we observed that the association of SBP with change in NT-proBNP was only significant in women, whereas the association with DBP was only significant in men, showing significant sex interaction. However, our study sample size does not allow for conclusions on sex differences and needs further investigation in larger cohorts. Aside from that, women received fewer beta-blockers and angiotensin-II receptor blockers than men, and non-invasive blood pressure is often underestimated in women, highlighting potential undertreatment in women.^21^ Furthermore, we observed sex differences in the development of both morphological and functional abnormalities. This is potentially explained by known sex differences in echocardiographic parameters such as E/e’ ratio and LAVI, which are not considered by the HFA-PEFF algorithm. But again, as the sample size is rather small, differences between men and women should be interpreted with caution.

### Early intervention in preclinical LVDD

While underpowered for evaluating intensified cardiovascular risk factor control, our findings suggest early intervention may slow LVDD progression. Importantly, our study lacked a randomised design or control group to best address therapeutic research questions. Up to now, few trials have investigated pharmacological intervention in patients with preclinical heart disease. Three trials recruiting patients with systolic dysfunction or elevated NT-proBNP succeeded in reducing mortality and HF development.^22^,^24^ On the other hand, in one imaging-guided trial focusing on patients with preclinical LVDD who were randomised to treatment with an ACE-inhibitor and beta-blocker, or standard care, no reduction in HF events was observed.^25^ This might be due to low adherence (43%) in this study, which recruited elderly individuals with risk factors for HF.^25^

### Detection of HF

We used a standardised approach to detect HF, encompassing clinical examination, exercise echocardiography and NT-proBNP measurements. However, this extensive diagnostic approach may not be feasible for early detection of HF in the community. Previously, the STOP-HF,^26^ PONTIAC,^24^ Vic-ELF^27^ and RED-CVD^28^ studies applied stepped screening strategies involving questionnaires, natriuretic peptide measurements, electrocardiography and echocardiography. These strategies were successful in detecting HF patients, prompting further considerations regarding the optimal stage for (preventive) treatment.

### Strengths and limitations

The strengths of our study include its novelty in terms of investigating sex-specific changes in biomarkers and functional and morphological markers of LVDD severity, employing a longitudinal design with repeated measures to minimise interindividual differences. Limitations include moderate sample size, resulting in insufficient power to draw conclusions about sex differences. Additionally, there is a risk of measurement bias, given measurement differences between institutes and single measurements of blood pressure, eGFR and NT-proBNP at baseline and follow-up. Furthermore, we are not able to compare newer echocardiographic parameters such as diastolic strain and ventricular untwisting rate, given insufficient frame rate at baseline since these echocardiograms were performed as part of routine clinical practice. Finally, the relatively low prevalence of comorbidities and the potential selection bias towards more healthy individuals may hinder the generalisability of our study.

### Future perspectives

Future studies should evaluate early intervention with drugs like SGLT-2 inhibitors or GLP-1 receptor agonists in individuals with preclinical LVDD, which show promise in HFpEF patients and have renoprotective properties.^29^ Additionally, anti-inflammatory drugs and strategies slowing cardiovascular ageing (eg, exercise) warrant exploration. Finally, proteomic approaches may offer insight into the underlying mechanisms of LVDD progression and its sex-specific aspects,^30^ facilitating targeted intervention for both women and men.

## Conclusions

Our study demonstrates that only a small proportion of women and men with preclinical LVDD develop incident HF over a roughly 5-year follow-up period. High blood pressure and decreased kidney function were associated with higher levels of NT-proBNP. This highlights the need to further explore cardiorenal protection as a method to prevent HFpEF.

## Supplementary material

10.1136/openhrt-2024-003105 online supplemental file 1

## Data Availability

Data are available on reasonable request.
