# Supplementary material for: Incident HFpEF and time-dependent changes in markers of LVDD severity in women and men with preclinical LVDD
Source: Open Heart. 2025 May 4;12(1):e003105. doi: 10.1136/openhrt-2024-003105 (PMC12049951; doi:10.1136/openhrt-2024-003105)
Supplement: online supplemental file 1 [file openhrt-12-1-s001.docx]

**SUPPLEMENTAL MATERIAL**

**Supplemental Table 1:** Percentage of missing values for all variables used in regression analyses

**Supplemental Table 2:** Baseline characteristics of the study base, and participants eligible for the follow-up study, stratified by sex

**Supplemental Table 3:** Characteristics of the patients that developed HFpEF

**Supplemental Table 4:** Additional echocardiography parameters at baseline and follow-up

**Supplemental Figure 1:** Flowchart of study participants.

**Supplemental Methods 1:** Echocardiography Methods

**Supplemental Table 1:** Percentage of missing values for all variables used in regression analyses

| **Variable** | **Percentage missing** |
| --- | --- |
| Age, baseline | 0.0 |
| Age, follow-up | 0.0 |
| Education level, baseline | 1.4 |
| Dyslipidemia, baseline | 0.0 |
| CVD history, baseline | 0.0 |
| alcohol, baseline | 5.0 |
| alcohol, follow-up | 5.5 |
| smoking, baseline | 1.4 |
| smoking, follow-up | 1.4 |
| BMI, baseline | 0.0 |
| BMI, follow-up | 0.0 |
| self-reported hypertension, baseline | 0.0 |
| self-reported hypertension, follow-up | 0.0 |
| diabetes, baseline | 0.0 |
| eGFR, baseline | 0.7 |
| eGFR, follow-up | 38.4 |
| SBP, baseline | 4.1 |
| SBP, follow-up | 0.0 |
| DBP, baseline | 4.1 |
| DBP, follow-up | 1.4 |
| Uncontrolled hypertension, baseline | 4.1 |
| Uncontrolled hypertension, follow-up | 0.0 |
| NT-proBNP, baseline | 0.7 |
| NT-proBNP, follow-up | 0.0 |
| Antihypertensive use, baseline | 0.0 |
| Antihypertensive use, follow-up | 0.0 |
| Number of antihypertensives used, baseline | 0.0 |
| Number of antihypertensives used, follow-up | 0.0 |
| E’ septal, baseline | 1.4 |
| E’ septal, follow-up | 0.7 |
| LAVI, baseline | 4.8 |
| LAVI, follow-up | 6.2 |
| Functional abnormalities HFA-PEFF score, baseline | 4.1 |
| Functional abnormalities HFA-PEFF score, follow-up | 0.0 |
| Morphological abnormalities HFA-PEFF score, baseline | 0.0 |
| Morphological abnormalities HFA-PEFF score, follow-up | 0.0 |

**Supplemental Table 2:** Baseline characteristics of the study base, and participants eligible for the follow-up study, stratified by sex

|  | Study base (n= 880) | | Eligible for follow-up (n= 213) | |
| --- | --- | --- | --- | --- |
|  | Men  (n= 276) | Women  (n= 604) | Men  (n=85) | Women (n=128) |
| Age, years, mean (±SD) | 63 (10) | 63 (9) | 64 (9) | 65 (9) |
| BMI, kg/m2, mean (±SD) | 27.3 (3.7) | 27.1 (4.8) | 27.7 (3.7) | 26.0 (4.4) |
| Obesity, n (%) | 53 (20) | 147 (25) | 14 ( 17) | 22 ( 18) |
| SBP, mmHg, mean (±SD) | 146 (17) | 142 (19) | 147 (18) | 143 (17) |
| DBP, mmHg, mean (±SD) | 88 (10) | 84 (10) | 89 (12) | 85 (10) |
| Creatinine, mmol/L, mean (±SD) | 81 (14) | 67 (11) | 79 (12) | 67 (11) |
| eGFR (CKD-epi), mL/min/1.73m2, mean (±SD) | 91 (13) | 88 (14) | 92 (11) | 86 (13) |
| eGFR (CKD-epi including cystatin C), mL/min/1.73m2, mean (±SD) | 87 (16) | 87 (16) | 89 (14) | 86 (15) |
| Self-reported hypertension, n (%) | 162 (58.7) | 342 (56.6) | 59 (69) | 69 (54) |
| Hypercholesterolemia, n (%) | 107 (39) | 255 (42) | 38 ( 45) | 60 (47) |
| Diabetes, n (%) | 28 (10) | 41 (7) | 12 (14) | 5 (4) |
| HbA1c, mmol/mol, mean (±SD) | 37 (7) | 37 (6) | 38 (9) | 36 (6) |
| Atrial fibrillation, n (%) | 7 (3) | 10 (2) | 3 (4) | 1 (1) |
| Ischemic heart disease, n (%) | 49 (18) | 49 (8) | 16 ( 19) | 10 (8) |
| Alcohol consumption, n (%) |  |  |  |  |
| No | 16 ( 6) | 87 (15) | 6 (7) | 12 (10) |
| Not daily | 107 (40) | 267 (47) | 36 (44) | 60 (50) |
| Daily | 145 (54) | 210 (37) | 40 (49) | 48 (40) |
| Smoking, n (%) |  |  |  |  |
| Never | 93 (34) | 249 (42) | 31 (37) | 56 (44) |
| Current | 29 (11) | 50 ( 8) | 10 (11.9) | 8 (6) |
| Former | 150 (55) | 301 (50) | 43 (51) | 62 (49) |
| Any anti-hypertensive use, n (%) | 110 (40) | 250 (41) | 36 ( 42) | 45 ( 35) |
| Beta-blockers | 31 (11) | 102 (17) | 10 ( 12) | 15 ( 12) |
| ACE-inhibitors | 46 (17) | 60 (10) | 15 (18) | 12 (9) |
| ARBs | 28 (10) | 71 (12) | 15 (18) | 12 (9) |
| CCBs | 22 ( 8) | 61 (10) | 8 (9) | 11 (9) |
| Thiazide diuretics | 43 (16) | 95 (16) | 11 ( 13) | 21 (16) |
| Statins, n (%) | 69 (25) | 118 (20) | 25 ( 29) | 29 (23) |
| Hypoglicemic agents, n (%) | 19 (7) | 26 ( 4) | 10 ( 12) | 3 (2) |
| LVEF, % (Teich), mean (±SD) | 67 (9) | 68 (8) | 67 (8) | 67 (8) |
| E velocity (cm/sec), mean (±SD) | 67 (16) | 71 (17) | 65 (16) | 69 (18) |
| E/A ratio, mean (±SD) | 0.97 (0.41) | 0.94 (0.30) | 0.89 (0.26) | 0.90 (0.24) |
| E’ lat, cm/sec, mean (±SD) | 8.9 (2.4) | 8.7 (4.6) | 8.0 (2.1) | 7.8 (2.0) |
| E’ lat < 10 cm/sec, n (%) | 158 (62) | 386 (66) | 67 (81) | 100 (81) |
| E’ sept, cm/sec, mean (±SD) | 7.2 (1.9) | 7.1 (3.9) | 6.5 (1.7) | 6.4 (1.6) |
| E’ sept < 7 cm/sec, n (%) | 109 (41) | 258 (44) | 48 (57) | 70 (56) |
| E/e’ ratio, mean (±SD) | 8.8 (2.7) | 9.5 (2.7) | 9.1 (2.2) | 9.9 (2.5) |
| E/e’ < 9, n (%) | 151 (59) | 274 (47) | 42 (51) | 46 (37) |
| E/e’ 9-14, n (%) | 101 (39) | 288 (49) | 39 (47) | 73 (59) |
| E/e’ ≥ 15, n (%) | 6 (2) | 21 (4) | 2 (2) | 4 (3) |
| Tricuspid regurgiration velocity, cm/sec, mean (±SD) | 242 (37) | 237 (26) | 235 (28) | 232 (19) |
| RWT, mean (±SD) | 0.43 (0.09) | 0.43 (0.08) | 0.47 (0.12) | 0.45 (0.08) |
| LVMI, g/m², mean (±SD) | 81 (22) | 71 (16) | 87 (24) | 73 (16) |
| LAVI, mL/m², g/m2, mean (±SD) | 26 (9) | 25 (10) | 27 (8) | 26 (9) |
| LAVI > 34 mL/m², mean (±SD) | 39 (15) | 79 (14) | 14 (17) | 19 (16) |
| LV Geometry, n (%) |  |  |  |  |
| Normal | 126 (48) | 273 (48) | 26 (33) | 48 (40) |
| Concentric remodeling | 116 (44) | 255 (45) | 44 (55) | 66 (56) |
| Concentric hypertrophy | 7 (3) | 16 (3) | 2 (3) | 0 (0) |
| Eccentric hypertrophy | 12 (5) | 24 (4) | 8 (10) | 5 (4) |
| NT-proBNP, pg/mL, median [25th quartile, 75th quartile] | 55 [36, 122] | 83 [54, 136] | 54 [29, 112] | 91 [54, 131] |
| NT-proBNP categories HFA-PEFF score, n (%) |  |  |  |  |
| Normal | 203 (76) | 428 (72) | 66 (79) | 91 (72) |
| Mildly elevated | 38 (14) | 101 (17) | 14 (17) | 25 (20) |
| Severely elevated | 28 (10) | 66 (11) | 4 (5) | 11 (9) |
| Functional abnormalities HFA-PEFF score, n (%) |  |  |  |  |
| absent | 62 (24) | 122 (21) | 5 (6) | 10 (8) |
| minor | 19 (7) | 50 (9) | 6 (7) | 7 (6) |
| major | 177 (69) | 411 (71) | 72 (87) | 106 ( 86) |
| Morphological abnormalities HFA-PEFF score, n (%) |  |  |  |  |
| absent | 99 (36) | 216 (36) | 12 (14) | 31 (24) |
| minor | 113 (41) | 287 (48) | 44 (52) | 74 (58) |
| major | 64 (23) | 101 (17) | 29 (34) | 23 (18) |
| ACC/AHA HF classification |  |  |  |  |
| stage A | 102 (37) | 193 (32) | 0 (0) | 0 (0) |
| stage B | 104 (38) | 158 (26) | 85 (100) | 128 (100) |
| stage C/D | 68 (25) | 251 (42) | 0 (0) | 0 (0) |
| Abbreviations: ACC/AHA HF classification refers to the American College of Cardiology/American Heart Association Heart Failure classification, stage A is defined as at high risk for HF but without structural or functional heart disease or symptoms of HF, stage B is defined as structural or functional heart disease but without signs or symptoms of H, and stage C/D is defined as structural or functional heart disease with prior or current symptoms of HF / Refractory HF requiring specialized interventions. ACE: angiotensin converting enzyme. AF: atrial fibrillation. ARBs: angiotensin receptor blockers. BMI: body mass index. CAD: coronary artery disease. CCB: calcium-channel blockers. DBP: diastolic blood pressure. eGFR: estimated glomerular filtration rate. HbA1c: glycated hemoglobin. HFA-PEFF refers to the diagnostic HFpEF algorithm by the Heart Failure Association from the European Society of Cardiology. LAVI: left atrial volume index. SBP: systolic blood pressure. LVEF: left ventricular ejection fraction. LVMI: left ventricular mass index. NT-proBNP: N-terminal pro-brain natriuretic peptide. RWT: relative wall thickness. | | | | |

**Supplemental Table 3:** Characteristics of the patients that developed HFpEF

| HFpEF patients (n=13), women (70%) | | |
| --- | --- | --- |
|  | Baseline | Follow-up |
| Age, years, mean (±SD) | 67 (8) | 71 (8) |
| BMI, kg/m2, mean (±SD) | 27 (5) | 28 (6) |
| Obesity, n (%) | 2 (15) | 3 (23) |
| SBP, mmHg, mean (±SD) | 148 (20) | 152 (19) |
| DBP, mmHg, mean (±SD) | 84 (12) | 79 (14) |
| Creatinine, mmol/L, mean (±SD) | 72 (12) | 84 (21) |
| eGFR (CKD-epi), mL/min/1.73m2, mean (±SD) | 86 (12) | 73 (18) |
| eGFR (CKD-epi including cystatin C), mL/min/1.73m2, mean (±SD) | 81 (13) | 68 (20) |
| Proportion of predicted workload achieved, mean (±SD) | 0.84 (0.20) | 0.96 (0.24) |
| Self-reported hypertension, n (%) | 7 (54) | 10 (77) |
| Hypercholesterolemia, n (%) | 6 (46) | 5 (39) |
| Diabetes, n (%) | 1 (8) | 1 (8) |
| HbA1c, mmol/mol, mean (±SD) | 37 (3) | 39 (6) |
| Atrial fibrillation, n (%) | 0 (0) | 1 (8) |
| Ischemic heart disease, n (%) | 0 (0) | 0 (0) |
| Alcohol consumption, n (%) |  |  |
| No | 1 (8) | 1 (8) |
| Not daily | 6 (50) | 6 (50) |
| Daily | 5 (42) | 5 (42) |
| Smoking, n (%) |  |  |
| Never | 5 (39) | 5 (39) |
| Current | 1 (8) | 0 (0) |
| Former | 7 (54) | 8 (62) |
| Any anti-hypertensive use, n (%) | 6 (46) | 11 (85) |
| Beta-blockers | 1 (8) | 2 (15) |
| ACE-inhibitors | 1 (8) | 1 (8) |
| ARBs | 2 (15) | 6 (46) |
| CCBs | 1 (8) | 3 (23) |
| Thiazide diuretics | 2 (15) | 3 (23) |
| Statins, n (%) | 4 (30.8) | 6 (46.2) |
| Hypoglicemic agents, n (%) | 0 (0) | 1 (8) |
| LVEF, % (Teich), mean (±SD) | 68 (6) | 65 (9) |
| E velocity (cm/sec), mean (±SD) | 65 (12) | 59 (13) |
| E/A ratio, mean (±SD) | 0.81 (0.22) | 0.78 (0.29) |
| E’ lat, cm/sec, mean (±SD) | 7.3 (1.8) | 7.2 (1.8) |
| E’ lat < 10 cm/sec, n (%) | 11 (92) | 12 (92) |
| E’ sept, cm/sec, mean (±SD) | 5.7 (1.4) | 5.4 (1.8) |
| E’ sept < 7 cm/sec, n (%) | 10 (77) | 8 (62) |
| E/e’ ratio, mean (±SD) | 10.0 (2.8) | 10.0 (3.5) |
| E/e’ < 9, n (%) | 6 (50) | 7 (54) |
| E/e’ 9-14, n (%) | 5 (42) | 4 (31) |
| E/e’ ≥ 15, n (%) | 1 (8) | 2 (15) |
| Tricuspid regurgiration velocity, cm/sec, mean (±SD) | NA | 217 (29) |
| RWT, mean (±SD) | 0.50 (0.15) | 0.51 (0.08) |
| LVMI, g/m², mean (±SD) | 87 (39) | 83 (28) |
| LAVI, mL/m², g/m2, mean (±SD) | 23 (6) | 36 (12) |
| LAVI > 34 mL/m², mean (±SD) | 1 (8) | 9 (70) |
| LV Geometry, n (%) |  |  |
| Normal | 4 (31) | 4 (31) |
| Concentric remodeling | 7 (54) | 7 (54) |
| Concentric hypertrophy | 2 (15) | 2 (15) |
| Eccentric hypertrophy | 0 (0) | 0 (0) |
| NT-proBNP, pg/mL, median [25th quartile, 75th quartile] | 120 [91, 161] | 222 [151, 451] |
| NT-proBNP categories HFA-PEFF score, n (%) | |  |
| Normal | 7 (54) | 3 (23) |
| Mildly elevated | 5 (39) | 3 (23) |
| Severely elevated | 1 (8) | 7 (54) |
| Functional abnormalities HFA-PEFF score, n (%) | |  |
| absent | 1 (8) | 0 (0) |
| minor | 6 (50) | 0 (0) |
| major | 5 (39) | 13 (100) |
| Morphological abnormalities HFA-PEFF score, n (%) | |  |
| absent | 3 (23) | 2 (15) |
| minor | 7 (54) | 3 (23) |
| major | 3 (23) | 8 (62) |
| Abbreviations: ACE: angiotensin converting enzyme. AF: atrial fibrillation. ARBs: angiotensin receptor blockers. BMI: body mass index. CAD: coronary artery disease. CCB: calcium-channel blockers. DBP: diastolic blood pressure. eGFR: estimated glomerular filtration rate. HbA1c: glycated hemoglobin. HFA-PEFF refers to the diagnostic HFpEF algorithm by the Heart Failure Association from the European Society of Cardiology. LAVI: left atrial volume index. SBP: systolic blood pressure. LVEF: left ventricular ejection fraction. LVMI: left ventricular mass index. NT-proBNP: N-terminal pro-brain natriuretic peptide. RWT: relative wall thickness. | | |

**Supplemental Table 4:** Additional echocardiography parameters at baseline and follow-up

|  | **Overall (n= 146)** | | **Men (n=61)** | | **Women (n= 85)** | |
| --- | --- | --- | --- | --- | --- | --- |
|  |  |  |  |  |  |  |
|  | **Baseline** | **Follow-up** | **Baseline** | **Follow-up** | **Baseline** | **Follow-up** |
| LVEF, % (Teich), mean (±SD) | 67 (7) | 66 (12) | 67 (8) | 65 (12) | 67 (7) | 67 (12) |
| LVEF, % (2D or 3D), mean (±SD) |  | 53 (31) |  | 53 (28) |  | 53 (34) |
| E velocity (cm/sec), mean (±SD) | 68 (18) | 61 (15) | 64 (17) | 61 (17) | 70 (18) | 60 (13) |
| E/A ratio, mean (±SD) | 0.92 (0.25) | 0.86 (0.22) | 0.93 (0.28) | 0.88 (0.25) | 0.91 (0.23) | 0.84 (0.20) |
| E’ lat, cm/sec, mean (±SD) | 8.1 (2.2) | 8.1 (2.1) | 8.4 (2.3) | 8.6 (2.0) | 8.0 (2.1) | 7.7 (2.1) |
| E’ lat < 10 cm/sec, n (%) | 111 (78) | 114 (78) | 44 (75) | 41 (67) | 67 (81) | 73 ( 86) |
| E’ sept, cm/sec, mean (±SD) | 6.7 (1.7) | 5.9 (1.5) | 6.7 (1.7) | 6.3 (1.5) | 6.7 (1.6) | 5.5 (1.5) |
| E’ sept < 7 cm/sec, n (%) | 71 (49) | 96 (66) | 31 (52) | 32 (53) | 40 (48) | 64 (76) |
| E/e’ ratio, mean (±SD) | 9.2 (2.3) | 9.1 (2.8) | 8.7 (1.9) | 8.3 (2.2) | 9.6 (2.5) | 9.6 (3.1) |
| E/e’ < 9, n (%) | 68 (49) | 86 (59) | 33 (56) | 40 (66) | 35 (43) | 46 (54) |
| E/e’ 9-14, n (%) | 68 (49) | 52 (36) | 25 (42) | 20 (33) | 43 (53) | 32 (38) |
| E/e’ ≥ 15, n (%) | 4 (3) | 8 (6) | 1 (2) | 1 (2) | 3 (4) | 7 (8) |
| Tricuspid regurgiration velocity, cm/sec, mean (±SD) | 228 (19) | 229 (30) | 226 (21) | 229 (31) | 226 (19) | 230 (29) |
| RWT, mean (±SD) | 0.45 (0.11) | 0.44 (0.09) | 0.47 (0.13) | 0.45 (0.09) | 0.44 (0.09) | 0.44 (0.09) |
| LVMI, g/m², mean (±SD) | 78 (21) | 75 (20) | 88 (26) | 83 (22) | 71 (13) | 70 (16) |
| LAVI, mL/m², g/m2, mean (±SD) | 26.6 (8.0) | 31.1 (8.9) | 27.8 (7.5) | 33.2 (9.6) | 25.7 (8.3) | 29.5 (8.0) |
| LAVI > 34 mL/m², mean (±SD) | 25 (18.0) | 48 (35.0) | 12 (20.3) | 24 (40.7) | 13 (16.2) | 24 (30.8) |
| LV Geometry, n (%) |  |  |  |  |  |  |
| Normal | 55 (40) | 60 (44) | 19 (33) | 24 (43) | 36 (46) | 36 (45) |
| Concentric remodeling | 73 (53) | 68 (50) | 30 (52) | 29 (52) | 43 (54) | 39 (49) |
| Concentric hypertrophy | 8 (6) | 2 (2) | 8 (14) | 1 (2) | 0 (0.0) | 1 (1) |
| Eccentric hypertrophy | 1 (1) | 6 (4) | 1 (2) | 2 (4) | 0 (0.0) | 4 (5) |
| NT-proBNP categories HFA-PEFF score, n (%) | |  |  |  |  |  |
| Normal | 112 (77) | 92 (63) | 47 (78) | 42 (69) | 65 (76) | 50 (59) |
| Mildly elevated | 25 (17) | 33 (23) | 10 (17) | 11 (18) | 15 (18) | 22 (26) |
| Severely elevated | 8 (6) | 21 (14) | 3 (5) | 8 (13) | 5 (6) | 13 (15) |
| LAVI: left atrial volume index. LVEF: left ventricular ejection fraction. LVMI: left ventricular mass index. NT-proBNP: N-terminal pro-brain natriuretic peptide. RWT: relative wall thickness. | | | | | | |

**Supplemental Figure 1:** Flowchart of study participants.


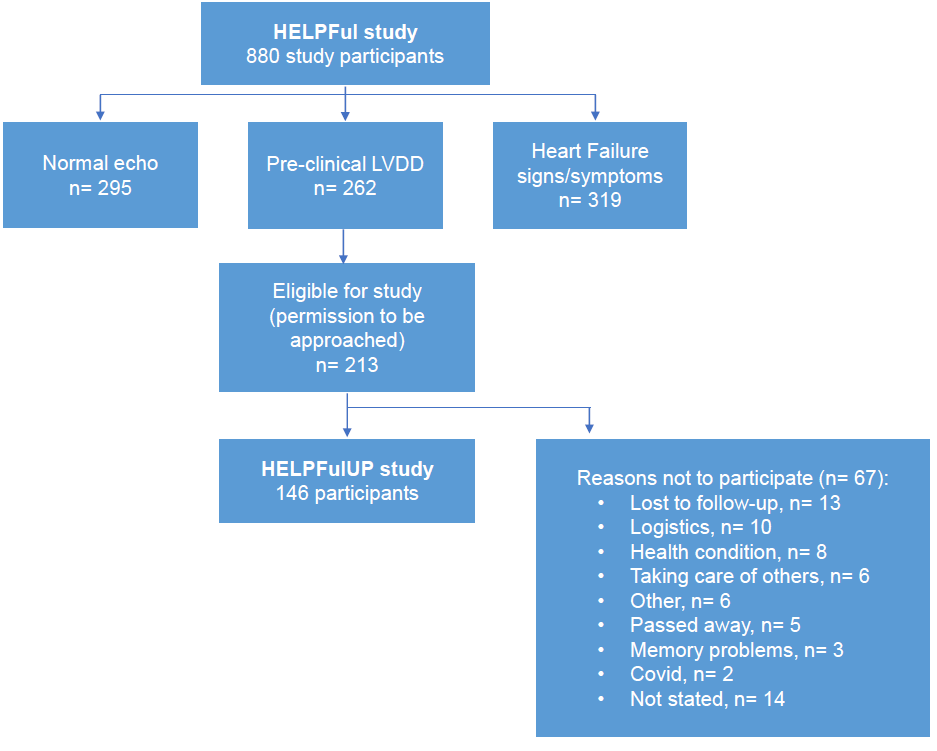


**Supplemental Methods 1:** Echocardiography Methods

**Baseline echocardiography**

Comprehensive transthoracic echocardiographic examinations at baseline were performed at the Cardiology Centers of the Netherlands (Utrecht location) by trained sonographers and interpreted by a cardiologist in accordance with the European Association of Cardiovascular Imaging 2016 recommendations for chamber quantification (1,2). The left ventricular ejection fraction (LVEF) was assessed quantitatively (Teichholz), or semi-quantitatively (eye-balling). Multiple diastolic parameters were measured, including pulsed-wave Doppler of the mitral and pulmonary venous inflow and tissue Doppler imaging of the mitral annulus motion. The ratio of peak early (E) diastolic filling velocity to peak atrial (A) contraction filling velocity was calculated to derive the E/A ratio. The early diastolic mitral annular recoil velocity (e’) was determined at both the septal and lateral wall. The E/e’ ratio was calculated by dividing E with the average of septal and lateral e’. LAVI was derived from tracing the left atrium during maximal atrial filling in the apical two-chamber and apical four-chamber views and subsequently indexing by body surface area (BSA). LVMI was calculated according to the formula validated by Devereux and subsequently indexed by BSA (3). The sonographers assessed TR in the parasternal right ventricular inflow, parasternal short- axis and apical four-chamber views. A minimum of five sequential complexes were recorded. The peak velocity of the TR signal was measured with continuous-wave Doppler and the systolic pulmonary artery pressure was calculated with the modified Bernoulli's equation. No strain measurements were performed at baseline.

**Follow-up echocardiography**

At follow-up echocardiography was performed by A.v.O. or E.D.C. at the University Medical Center Utrecht using a General Electric Vivid E95 device (Horten, Norway). The protocol for rest echocardiography was similar to baseline, but the preferred method to assess LVEF differed. LVEF was calculated from LV end-diastolic and end-systolic volume estimates derived from 3D or 2D echocardiography (biplane method of disks, i.e. modified Simpson’s rule). Additionally, high frame rate images of the left ventricle and left atrium were acquired to measure global longitudinal strain.

**Exercise echocardiography**

At follow-up, all participants additionally underwent stepwise incremental supine bicycle exercise-echocardiography (Lode Angio, Groningen, The Netherlands) targeted to 70% of predicted workload in approximately 15 minutes, unless limited by complaints or exhaustion (4). This bicycle was tilted leftwards to obtain optimal image quality. Predicted workload was calculated according to the sex-specific formula proposed by Jones (5). During image acquisition the workload was set to a fixed level of 25%, 50% and 70% of the predicted workload.

A pre-set exercise imaging protocol was used, as provided by GE software. We acquired images at three stages during exercise and during recovery. Measurements included e’ velocities, E/e’ ratio, TR velocity and left ventricular outflow tract V1 velocity to calculate cardiac output. In order to only record adequate measures, we considered E/A fusion and image quality. If these criteria were not met, the data was reported missing.

**References:**

1. Valstar GB, Bots SH, Groepenhoff F, Gohar A, Rutten FH, Leiner T, et al. Discovery of biomarkers for the presence and progression of left ventricular diastolic dysfunction and HEart faiLure with Preserved ejection Fraction in patients at risk for cardiovascular disease: Rationale and design of the HELPFul case-cohort study in . BMJ Open. 2019;9(6):1–8.

2. Lang RM, Badano LP, Mor-Avi V, Afilalo J, Armstrong A, Ernande L, et al. Recommendations for Cardiac Chamber Quantification by Echocardiography in Adults: An Update from the American Society of Echocardiography and the European Association of Cardiovascular Imaging. Journal of the American Society of Echocardiography [Internet]. 2015 Jan;28(1):1-39.e14. Available from: https://linkinghub.elsevier.com/retrieve/pii/S0894731714007457

3. Devereux RB, Alonso DR, Lutas EM, Gottlieb GJ, Campo E, Sachs I, et al. Echocardiographic assessment of left ventricular hypertrophy: Comparison to necropsy findings. Am J Cardiol. 1986;57(6):450–8.

4. La Gerche A, Claessen G, Van De Bruaene A, Pattyn N, Van Cleemput J, Gewillig M, et al. Cardiac MRI: A new gold standard for ventricular volume quantification during high-intensity exercise. Circ Cardiovasc Imaging. 2013;6(2):329–38.

5. Jones N. Clinical Exercise Testing. 1997th ed. Philadephia: WB Saunders; 1997.
